# Mechanosensitive caveolin-1 activation-induced PI3K/Akt/mTOR signaling pathway promotes breast cancer motility, invadopodia formation and metastasis *in vivo*

**DOI:** 10.18632/oncotarget.7583

**Published:** 2016-02-22

**Authors:** Hong Yang, Liuyuan Guan, Shun Li, Ying Jiang, Niya Xiong, Li Li, Chunhui Wu, Hongjuan Zeng, Yiyao Liu

**Affiliations:** ^1^ Department of Biophysics, School of Life Science and Technology of China, Chengdu 610054, Sichuan, P.R. China; ^2^ Center for Information in Biomedicine, University of Electronic Science and Technology of China, Chengdu 610054, Sichuan, P.R. China

**Keywords:** low shear stress, caveolin-1, PI3K/Akt/mTOR, cell motility, invadopodia formation

## Abstract

Cancer cells are subjected to fluid shear stress during passage through the venous and lymphatic system. Caveolin-1 (Cav-1), a principal structural component of caveolar membrane domains, contributes to cancer development but its mechanobiological roles under low shear stress (LSS) conditions remain largely unknown. Here, we identified Cav-1 is mechanosensitive to LSS exposure, and its activation-induced PI3K/Akt/mTOR signaling promotes motility, invadopodia formation and metastasis of breast carcinoma MDA-MB-231 cells. Application of LSS (1.8 and 4.0 dynes/cm^2^) to MDA-MB-231 cells significantly increased the cell motility, invadopodia formation, MT1-MMP expression, ECM degradation, and also induced a sustained activation of Cav-1 and PI3K/Akt/mTOR signaling cascades. Methyl-β-cyclodextrin-caused caveolae destruction markedly decreased LSS-induced activation of both Cav-1 and PI3K/Akt/mTOR, leading to suppress MT1-MMP expression, inhibit invadopodia formation and ECM degradation, suggesting that caveolae integrity also involved in metastasis. Immunocytochemical assay showed that LSS induces the Cav-1 clustering in lipid rafts and co-localization of Cav-1 and MT1-MMP on invadopodia. Immunofluorescence confocal analysis demonstrated that Cav-1 activation were required for the acquisition of a polarized phenotype in MDA-MB-231 cells. Finally, Cav-1 knockdown significantly suppressed tumor colonization in the lungs and distant metastases in animal models. Our findings highlight the importance of Cav-1 in hematogenous metastasis, and provide new insights into the underlying mechanisms of mechanotransduction induced by LSS.

## INTRODUCTION

Metastasis is the final stage in tumor progression and is thought to be responsible for up to 90% of deaths associated with solid tumors, which arises through a complex series of sequential steps [1]. During metastasis, some cancer cells escape from the primary tumor, enter the bloodstream and become circulating tumor cells in peripheral blood. Some of these circulating tumor cells acquire the capability to extravasate and colonize secondary sites, spreading tumors to distant organs. Metastasis occurs primarily in the venous circulation. This relatively low shear stress (LSS) venous environment facilitates the arrest of tumor cells to the vessel wall, eventually leading to penetration of the vascular wall. Therefore, identifying the precise molecular mechanisms which mediate cancer cell migration is necessary for us to design specific treatment to prevent breast cancer metastasis.

Increasing a number of evidence indicate that fluid shear stress plays a critical role in hematogenous metastasis. Circulating tumor cells are continuously exposed to shear stress in the bloodstream. The role of flow-induced mechanical forces in modulating cancer pathophysiology has just emerged [2]. However, how the circulating tumor cells perceive the shear stress and its downstream signal pathways are not well understood. Abundant evidence has accumulated that cholesterol- and sphingolipid-enriched membrane patches, termed lipid rafts, are important in signal transduction [3]. Caveolae as the macromolecular vesicular transporters, while their unique lipid composition classifies them as plasma membrane lipid rafts, structures enriched in a variety of signaling molecules. Recent evidence has accumulated suggesting that caveolae provides a platform where signals are processed that are essential to tumor cell growth, resistance to apoptotic stimuli, and other aggressive characteristics of cancer cells [4]. Caveolin-1 (Cav-1), a major component of caveolae, has been reported to participate in various cellular processes including, lipid transport, membrane trafficking, gene regulation, signal transduction and tumorigenesis [5]. Currently, great attention has been attracted in the study of the association of Cav-1 to tumor development and metastasis. In cancer models including prostate, breast and colon, Cav-1 expression is known to be upregulated and correlates with tumor progression and metastasis [6, 7]. Under normal conditions, Cav-1 has been shown to be phosphorylated in response to cytokines, and activation of the pathway in Cav-1-positive cells resulted in activation of other intracellular pathways [8]. For cancer cells, this capacity will be favored by the activation of signaling pathways that trigger the metastatic cascades.

Another the key step in the metastatic cascade involves the disruption of the extracellular matrix (ECM) and basement membranes, permitting tumor cells to access a distant metastatic site. Tumor cells are capable of expressing ECM-degrading enzymes. The matrix metalloproteinases (MMPs), being major components of the basement membranes, enable tumor cells to tissues in this region [9]. MMPs have been implicated in many aspects of cancer progression, such as tumor growth, angiogenesis, invasion, and metastasis [10, 11]. Among MMPs, membrane type 1-MMP (MT1-MMP) mainly involved in ECM degradation at the leading edge of invasive cancer cells [12–14]. In addition, formation of invadopodia allows cells to coordinate ECM degradation, thereby facilitating cell migration and invasion [15–17]. Invadopodia are unique protrusions observed at the cell adhesion sites of invasive cancer cells that are rich in cell-ECM adhesion molecules, actin assembly regulators, membrane-remodeling proteins, tyrosine kinases, tyrosine-phosphorylated proteins, and MMPs [10, 18, 19]. The matrix degradation activity at invadopodia rests on the accumulation and activation of MT1-MMP. To date, the mechanism by which cancer cells recognize changes in shear stress (mechanosensing) and control activation of multiple signaling pathways in a well-orchestrated manner is not well understood. Several potential mechanosensing systems have been proposed including cytoskeleton/integrins, G proteins, and ion channels [20, 21]. However, the specific roles of Cav-1 in migration of circulating tumor cells during hematogenous metastasis requires further investigation.

Here, we hypothesized that Cav-1 in the cell surface may be critical in mechanosensing and subsequent mechanosensitive cell signaling. In the present study, we provide evidence demonstrating the critical functional role of Cav-1 in promoting LSS-induced invadopodia formation and tumor metastases. LSS exposure was found to induce Cav-1 activation and PI3K/Akt/mTOR signaling cascade and to increase MT1-MMP expression and trafficking, cytoskeleton reorganization, invadopodia formation and ECM degradation, leading to promotion of tumor cell motility and metastasis. These improved understanding of metastasis and identifying key regulators could have important therapeutic implications.

## RESULTS

### LSS induces cell motility, invadopodia formation and gelatin degradation

**Figure 1:**
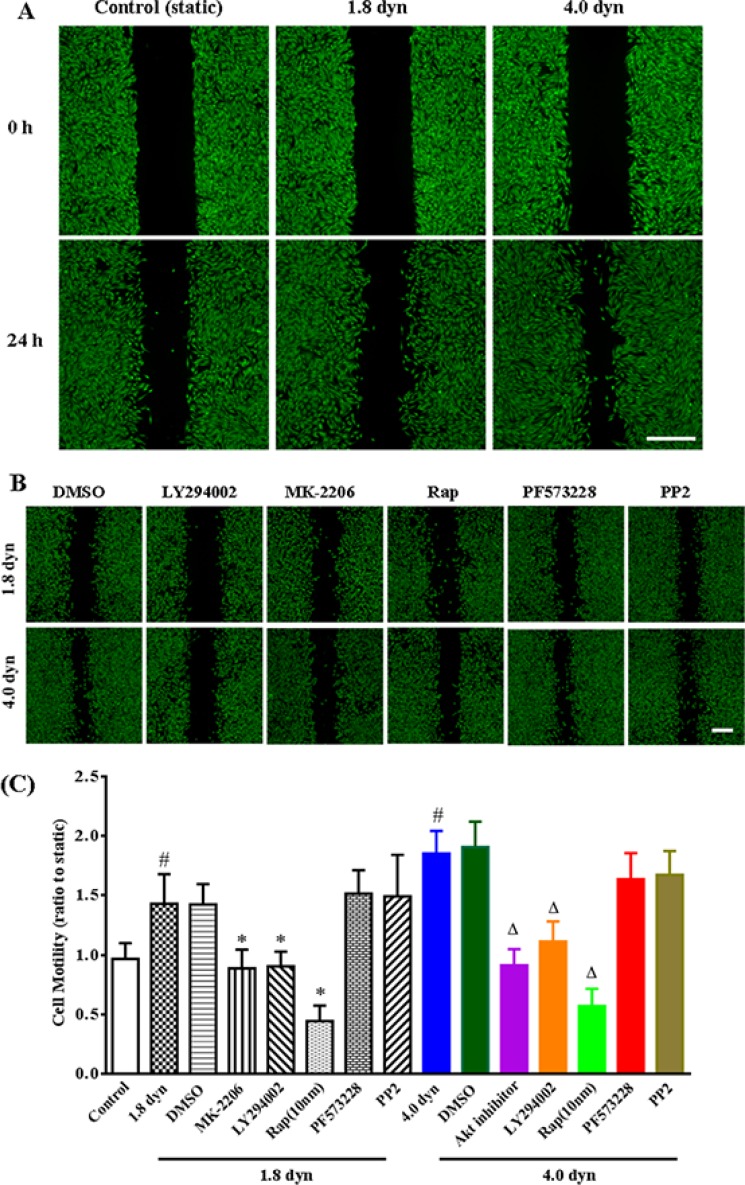
Evaluation of low shear stress (LSS) on cell motility and the roles of PI3K/Akt/mTOR pathway in cell motility by wound healing assay (**A**) The confluent MDA-MB-231 cell monolayers were wounded by scraping and treated with low shear stresses (1.8 and 4.0 dyn/cm^2^) for 1 h. Cell migration to the wound surface was monitored from 0 to 24 h, and stained by calcein-AM. The relative wound closure was observed under microscope and photographed. Scale bar = 200 μm. (**B**) LSS-induced cell motility assay after MDA-MB-231 cells were pretreated with inhibitors of PI3K (10 μM LY294002), Akt (20 μM MK-2206), mTOR (10 μM rapamycin, Rap), FAK (10 μM PF573228) or Src (10 μM PP2). Scale bar = 200 μm. (**C**) Quantification of relative closure of the scratch would was determined via calculating the wound area at six randomly selected fields by ImageJ software (NIH, USA). All the quantitative data were represented as mean ± SD. *p* < 0.05 was considered statistically significant (^#^*p* < 0.05 compared to the control (static), ^*^compared to the DMSO or 1.8 dyn, ^Δ^ compared to the DMSO or 4.0 dyn).

To determine whether shear stress could increase the cancer cell motility, human breast carcinoma MDA-MB-231 cell monolayers were kept static conditions or exposed to LSS (1.8 and 4.0 dynes/cm^2^) for 1 h after wound scratched. Cell migration to the wound surface was monitored from 0 to 24 h, and stained by calcein-AM (green), then examined by an inverted fluorescence microscopy. Figure 1 shows the typical micrographs of cell motility to the wound areas. By scratch motility assay, the static cultured MDA-MB-231 cells exhibit only a limited wound closure activity. In contrast, the LSS-treated cells show acceleration of wound closure that could be observed after treatment for 24 h, and these cells form multiple archipelago-like sheets of cells stretching into the denuded area (Figure 1A). Notably, inhibitors of PI3K (LY294002), Akt (MK-2206) and mTOR (rapamycin) markedly decreased the LSS-induced wound closure activity. However, pretreatment with inhibitors of FAK (PF573228) and Src (PP2) has no effect on the LSS-induced cell motility (Figure 1B). It is suggested that PI3K, Akt and mTOR might be participated LSS-induced cell motility in an FAK and Src-independent manner. The results of the wound closure assays were quantitated through a visual analysis (Figure 1C). Following the exposure to LSS and wounding for 24 h, six distinct view fields per sample were randomly selected, and the wound area were calculated by ImageJ software. The quantitative data are well consistent with the aforementioned representative images.

**Figure 2:**
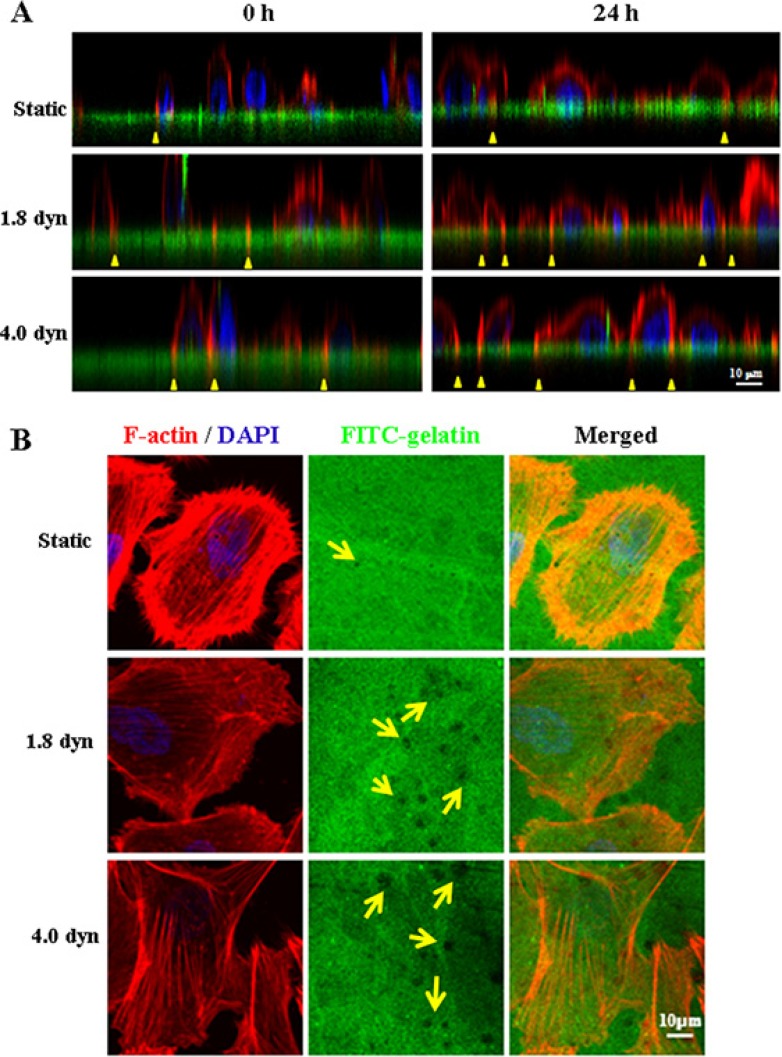
LSS induced invadopodia formation and ECM degradation MDA-MB-231 cells were cultured on fluorescent gelatin-coated coverslips, and then kept under static conditions as controls or subjected to low shear stress for 1 h. After LSS exposure, the cells were cultured for 24 h again, and stained for F-actin (red) and nucleus (blue) with TRTIC-conjugated phalloidin and DAPI, respectively. The invadopodia (yellow triangular arrows indicated) by 3D image reconstruction in *XZ* dimension (**A**) and gelatin degradation puncta (yellow arrows indicated) (**B**) were observed in *XY* dimension under a confocal microscope.

Tumor metastasis encompasses several processes, such as migration/invasion, extravasation and metastatic colonization. To further elucidate the effect of LSS on tumor metastasis, we investigated whether LSS exposure was correlated with the abilities of invadopodia formation and gelatin degradation. MDA-MB-231 cells were co-stained with F-actin by TRTIC-conjugated phalloidin (red) and DAPI (blue), and FITC-conjugated gelatin degradation assay to monitor invadopodial activity (gelatin degradation). As seen in Figure 2, the *z*-stack projection images (Figure 2A) showed more invasion of F-actin-rich invadopodia into the gelatin layer (green line) when the MDA-MB-231 cells were exposed to LSS, implying LSS exposure enhanced invadopodial formation (yellow triangular arrows indicated). Similar results were obtained in the *XY*-stack projection images, and more gelatin degradation puncta were observed in the LSS exposed cells (Figure 2B). These data indicated that MDA-MB-231 cells exposed to LSS promoted the invasion of F-actin into the gelatin layer and increased gelatin degradation as well.

### LSS facilitates Cav-1 clustering in lipid rafts

**Figure 3:**
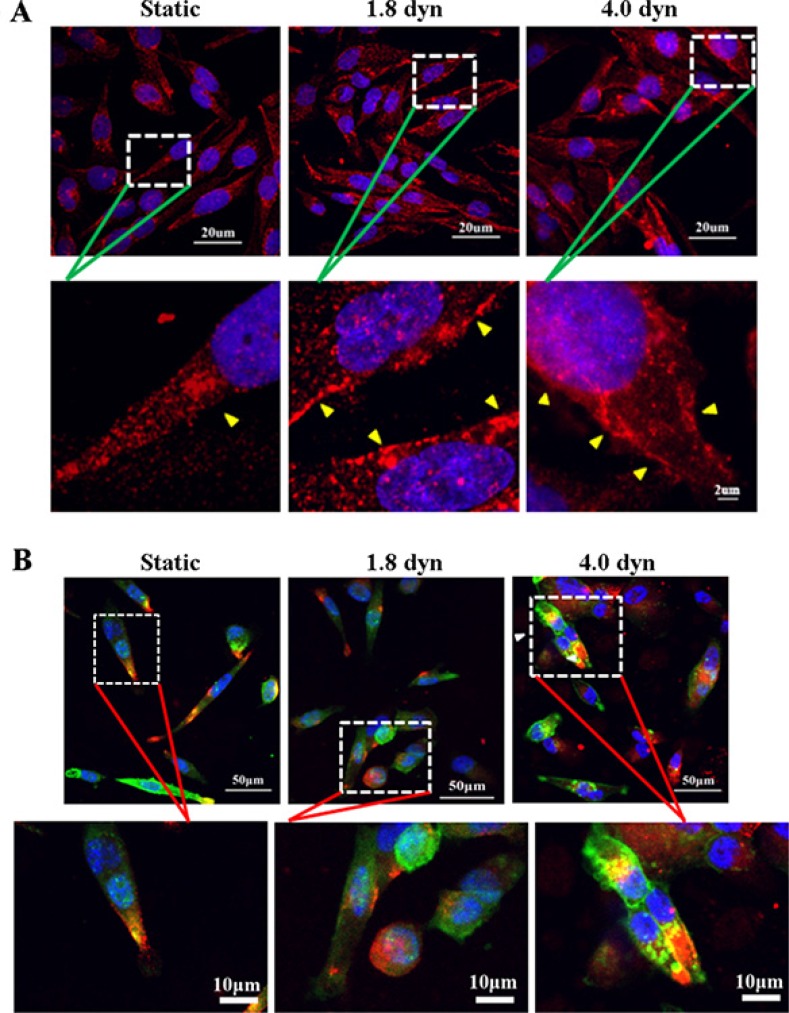
LSS induced Cav-1 translocation to cell membranes (yellow triangular arrows indicated) and localization on the lipid raft fraction MDA-MB-231 cells were kept under static condition as control or subjected to LSS for 1 h. Samples were stained with anti-caveolin-1 (red), whereas the lipid rafts (green) and nuclei (blue) were stained by Alexa Fluor 488-conjugated CTxB and DAPI, respectively. Immunofluorescent images were obtained under a confocal microscope to detect Cav-1 localization in cell membranes (**A**) or lipid raft (**B**).

**Figure 4:**
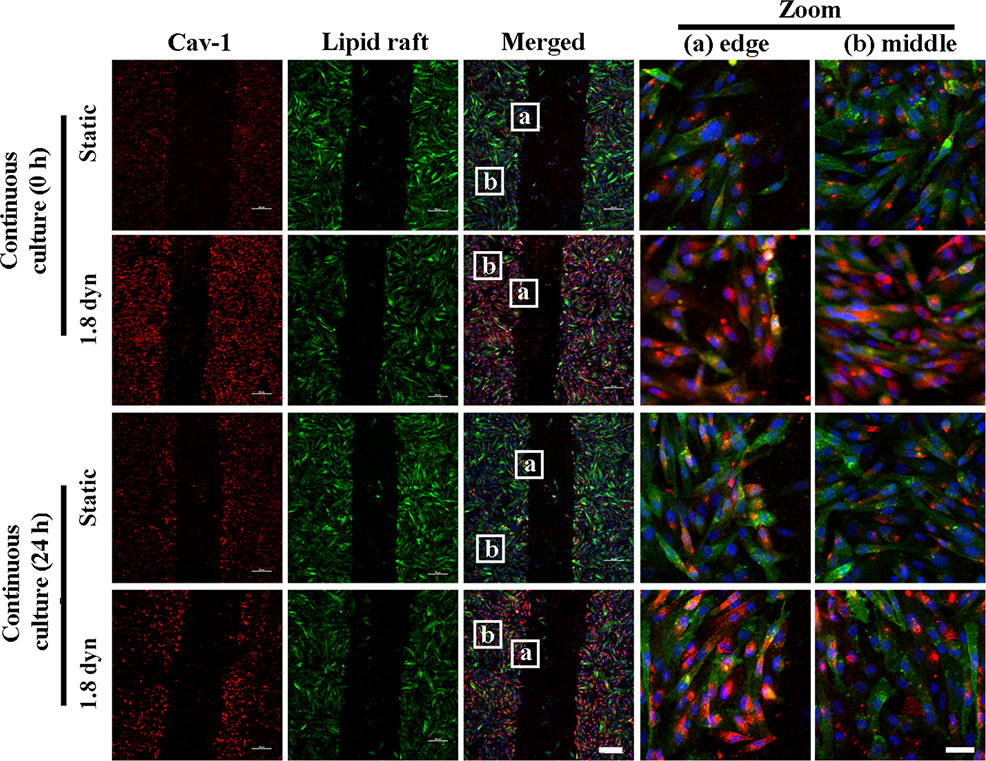
The differential Cav-1expression and localization on lipid raft in the scraped would edges (A) and cell monolayer middles (B) under LSS exposure MDA-MB-231 cells were kept under static condition as control or subjected to LSS (1.8 dyn/cm^2^) for 1 h. Samples were stained with anti-caveolin-1 (red), whereas the lipid rafts (green) were stained by Alexa Fluor 488-conjugated CTxB. Scale bar in merged = 100 μm, and scale bar in zoom = 10 μm.

Cav-1 is an essential component of caveolae, which are subtypes of lipid rafts, and is known to participate the organization and dynamics of lipid rafts [27, 32, 33]. Therefore, we wondered whether LSS changed Cav-1 distribution in cell membrane. Cav-1 was labeled by Texas red fluorescence (red). Results showed that Cav-1 preferentially localizes to the cytoplasm and cell membrane in static and sheared MDA-MB-231 cells, respectively (Figure 3A). However, it is still unknown that LSS-induced Cav-1 was clustered in lipid rafts or non-lipid rafts. To address and demonstrate this issue, Therefore, we further labeled the lipid rafts by using a lipid raft marker, CTxB, which binds to lipid raft-enriched GM1 ganglioside and has been widely exploited to visualize lipid rafts [34, 35]. Confocal microscopy data revealed that Cav-1 clustered on the lipid rafts of cell membrane after LSS exposure (Figure 3B). To better understand the role of Cav-1 clustering in lipid rafts in MDA-MB-231 cell motility, by scratch motility assay, we compared the distribution of Cav-1 in the wound-edge cells and beyond the wound-edge cells (middle) under LSS exposure (1.8 dyn, 1 h) or static condition. As showed in Figure 4, few Cav-1 in lipid rafts was observed in both wound-edge cells and middle cells at continuous culture for 0 or 24 h in static condition. Notably, abundant Cav-1 in lipid rafts was visualized in both wound-edge cells and middle cells after LSS exposure at continuous culture for 0 h. Of interest, only Cav-1 cluster in lipid rafts was detected in those wound-edge cells at continuous culture for 24 h after LSS exposure. Taken together, these results suggest that LSS could induce Cav-1 clustering in lipid rafts, and suggested that Cav-1 clustering in lipid rafts might correlate with cell motility capability.

### LSS activates Cav-1, upregulates Cav-1 and MT1-MMP expression, and promotes Cav-1/MT1-MMP co-localization in invadopodia

**Figure 5:**
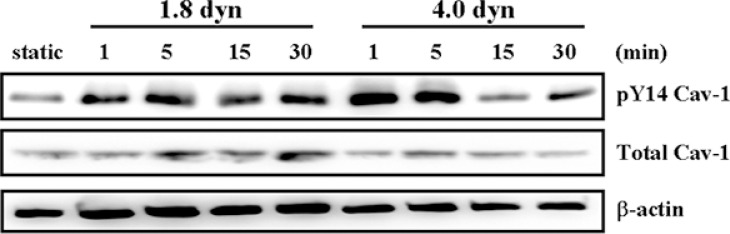
LSS induced Cav-1 activation MDA-MB-231 cells were kept under static condition as control or subjected to LSS for the indicated times. The phosphorylation of Cav-1 at Tyr 14 (pY14-Cav-1) was detected Western blot analysis.

**Figure 6:**
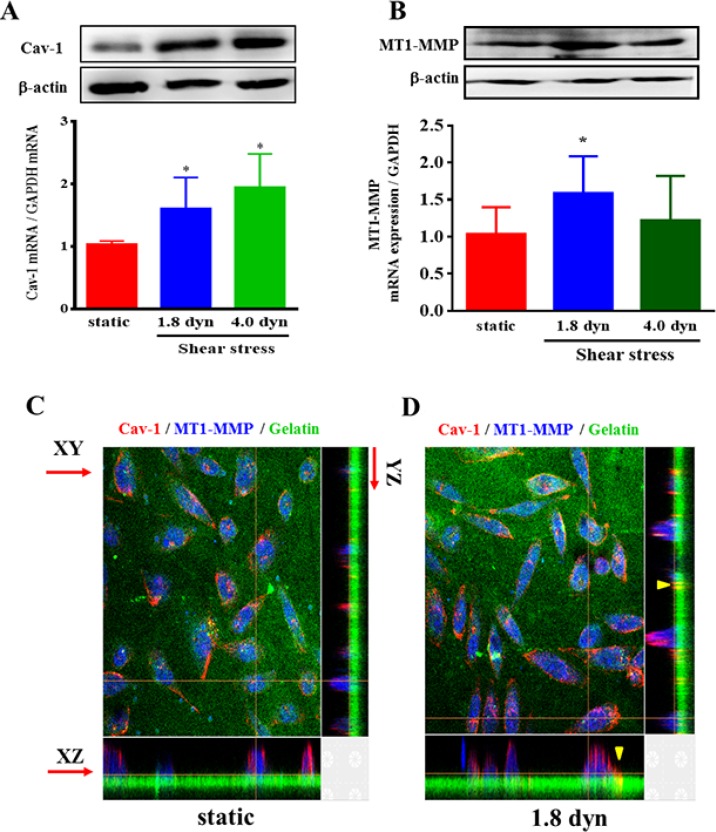
LSS increased Cav-1 and MT1-MMP expression and Cav-1/MT1-MMP co-localization in invadopodia MDA-MB-231 cells were kept under static condition as control or subjected to LSS for 1 h. After culture for 24 h, the Cav-1 (**A**) and MT1-MMP (**B**) expression were determined by Western blot analysis, and the Cav-1/MT1-MMP co-localization in invadopodia (yellow triangular arrows indicated) was observed under a confocal microscope in XZ and YZ dimensions (**C**, **D**). The cells were stained with anti-caveolin-1 (red), whereas the gelatin (green) and MT1-MMP (blue) were stained by FITC and DyLight 405, respectively. ^*^p < 0.05 shear stress *vs*. static.

Tyrosine phosphorylation on tyrosine 14 of Cav-1 (pY14 Cav-1) is a typical activation of Cav-1, which is crucial for regulation of multiple cancer-associated cellular processes [36, 37]. We further investigated the effects of LSS on Cav-1 activation in MDA-MB-231 cells. The cells were kept as controls or subjected to LSS (1.8 or 4.0 dyn) for 1, 5, 15, and 30 min, and Cav-1 phosphorylation was examined by using Western blot analysis. LSS applied to MDA-MB-231 cells induced rapid increases (significant within 5 min) in the Cav-1 phosphorylation (Figure 5). Previous studies reported that the expression level of Cav-1 and MT1-MMP showed to be associated with a poor prognosis and metastasis in several cancers [10, 38, 39]. Thus, we assessed whether LSS could affect Cav-1 and MT1-MMP expression. It was found that exposure of MDA-MB-231 cells to LSS (1.84 or 4.0 dyn, 1 h) resulted an increase of Cav-1 and MT1-MMP expression in both mRNA and protein levels (Figure 6A and 6B). The upregulation of Cav-1 and MT1-MMP expression would facilitate cancer cell migration. Because MT1-MMP in invadopodia was linked to the migratory activity of the cells, and caveolae could translocate the MT1-MMP to invadopodia. So the question of whether or not Cav-1/MT1-MMP are co-located at invadopodia might have important implications with regard to the mechanisms involved in the regulation of cancer cell invasion. Next, we further tested whether LSS exposure promotes Cav-1/MT1-MMP co-localization in invadopodia. Results showed that LSS (1.8 dyn, 1 h) promoted Cav-1/MT1-MMP co-localization in invadopodia (yellow triangular arrows indicated) (Figure 6C and 6D). Together, these results reveal a new mechanism that LSS enhances MDA-MB-231 cell motility through Cav-1 activation, Cav-1 and MT1-MMP expression upregulation, and Cav-1/MT1-MMP co-localization in invadopodia.

### Caveolae is required for gelatin degradation and LSS-induced activation of Cav-1 and PI3K/Akt signaling

**Figure 7:**
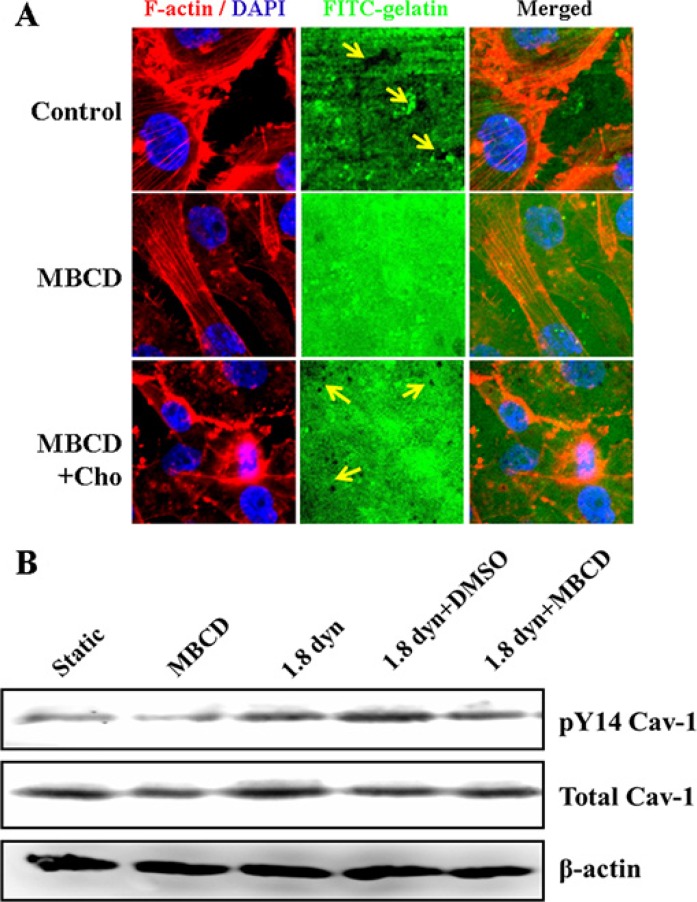
Caveolae is required for invadopodia formation (gelatin degradation) and LSS-induced Cav-1 activation in MDA-MB-231 cells (**A**) Evaluation of invadopodia activity by a fluorescent gelatin degradation assay under a confocal microscope. Cells were pretreated with MBCD (10 μM) for 1 h, and replaced by fresh cell culture medium. The gelatin degradation images were performed by plating cells in 24 h incubation. F-actin was stained with TRTIC-conjugated phalloidin (red), whereas nuclei (blue) were stained by DAPI. Arrows indicate gelatin degradation puncta. Scale bar = 10 μm. (**B**) MDA-MB-231 cells were pretreated with MBCD (10 μM) for 1 h, replaced by fresh cell culture medium, and followed by cholesterol replenishment (84 μg/mL) or not for 30 min. The cells were kept under static condition as control or subjected to low shear stress for 1 h. The phosphorylation of Cav-1 at Tyr 14 (pY14-Cav-1) was detected Western blot analysis.

Caveolae are specialized plasma membrane subdomains capable of transport and sophisticated compartmentalization of cell signaling. Numerous cell functions involve caveolae and require Cav-1 [40]. To determine whether caveolae is required for gelatin degradation in MDA-MB-231 cells, we plated the cells on FITC-gelatin. Gelatin matrix degradation is visualized as loss of green fluorescence background and cells were stained with TRTIC-conjugated phalloidin (red) and DAPI (blue). When the cells were pretreated with the cholesterol binding agent MBCD, known to disrupt lipid rafts (caveolae) by depleting cholesterol, for 1 h, we found that the cells with MBCD pretreatment showed a significant decrease in gelatin degradation activity (Figure 7A). However, cholesterol replenishment restored the destruction of caveolae induced by MBCD, and the gelatin degradation activity also markedly increased. More gelatin degradation puncta were observed (arrows marked). It suggested that caveolae participated the ECM degradation and invadopodia formation. Next, the role of caveolae in LSS-induced Cav-1 activation was further examined using Western blot assay. Pretreatment with MBCD inhibited Cav-1 activation, and also significantly abolished the LSS-induced activation of Cav-1 in MDA-MB-231 cells, as compared with the cells exposed with LSS alone (Figure 7B).

**Figure 8:**
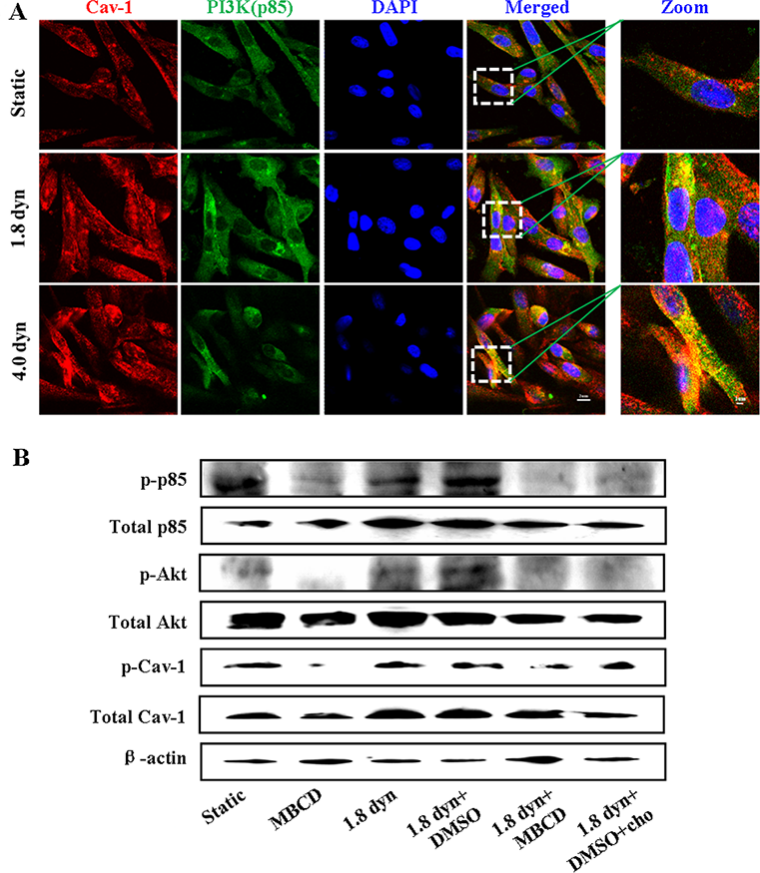
LSS-induced activation of the PI3K/Akt pathway MDA-MB-231 cells were kept under static condition as control or subjected to LSS for 1 h. The cells were stained with anti-caveolin-1 (red), whereas the PI3K (green) and nuclei (blue) were stained by anti-PI3K and DAPI, respectively. The co-localization of Cav-1 and PI3K was imaged under a confocal microscope (**A**). Scale bars in merged and zoon are 20 μm and 10 μm, respectively. (**B**) Caveolae is required for LSS-induced activation of the PI3K/Akt pathway. The phosphorylation of p85 (a regulatory subunit of PI3K), AKT, and Cav-1 induced by LSS or MBCD (10 μM) pretreatment was determined by Western blot analysis.

Previous studies have shown that activation of Cav-1 induces epithelial-to-mesenchymal transition of bladder cancer cells through upregulation of Slug expression, which occurs through activation of the PI3K/AKT signaling pathway [41]. To examine whether LSS could also induced activation of PI3K/AKT pathway as well as explore the role of caveolae in this process, we firstly investigated the co-localization of Cav-1 and p85 (a regulatory subunit of PI3K) in MDA-MB-231 cells. The cells were stained for Cav-1 (red) and nucleus (blue) with fluorescence labeled anti-Cav-1 and DAPI, respectively. LSS exposure dramatically increased the co-localization of Cav-1 and p85 (Figure 8A). p85 is normally cytosolic but translocates to cell membranes and the cytoskeleton or associated together with other signal molecules after PI3K activation, which has been used as a readout of PI3K activation [42, 43]. This observation implies that LSS can activate PI3K/Akt pathway. To further demonstrate this finding, their phosphorylation of PI3K, Akt (Ser473), and Cav-1 was examined by using Western blot analysis. MDA-MB-231 cells were pretreated with MBCD or exposed under LSS (1.8 dyn, 1 h). LSS applied to MDA-MB-231 cells induced an increase in the phosphorylation of these signal molecules, and pretreating MDA-MB-231 cells with the MBCD abolished LSS-induced their phosphorylation (Figure 8B). Altogether, these results indicate that caveolae and its integrity are required for gelatin degradation as well as in LSS-induced activation of Cav-1 and PI3K/Akt signaling.

### Cav-1 is necessary for MT1-MMP expression, PI3K/Akt signaling and LSS-induced Cav-1/MT1-MMP co-localization in cell membranes

**Figure 9:**
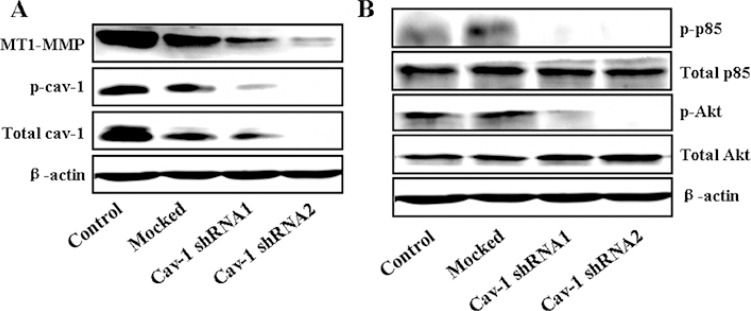
MT1-MMP expression and activation of the PI3K/Akt pathway required Cav-1 In MDA-MB-231 cells, reduction of Cav-1 by shRNA inhibited the MT1-MMP expression (**A**) and inhibited the PI3K/Akt pathway (**B**) by Western blot analysis.

**Figure 10:**
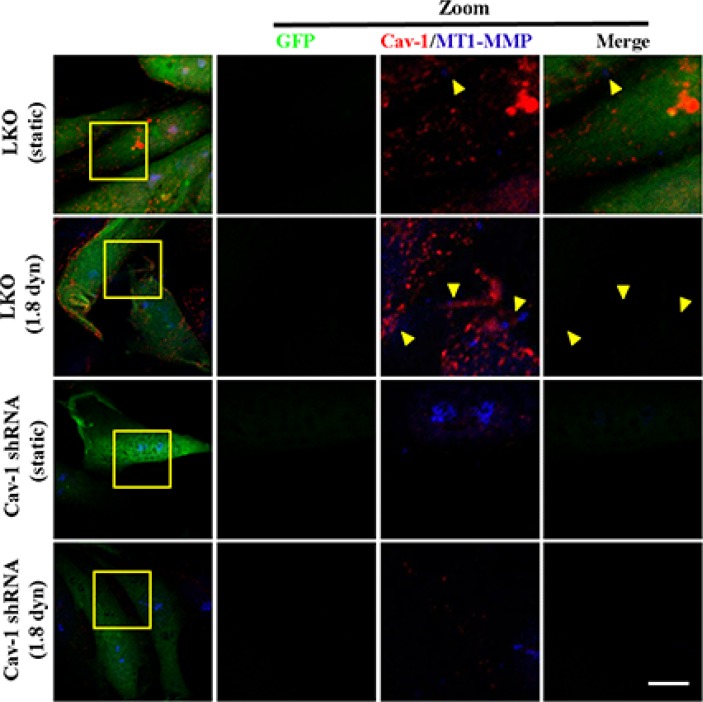
Cav-1 silence inhibited the LSS induced-colocalization of Cav-1 and MT1-MMP on the cell membrane MDA-MB-231 cells stably transfected with scrambled shRNA (LKO) or Cav-1 shRNA were kept under static condition as control or subjected to low shear stress (1.8 dyn) for 1 h. The samples were stained with anti-caveolin-1 and DyLight 405 to label the Cav-1 (red) MT1-MMP (blue), respectively, and observed under a confocal microscopy. Representative images from three independent experiments are shown. The right three panels are the magnification of the indicated areas in the left panel. Scale bar = 5 μm.

Cav-1 is a mediator of vesicular transport, cholesterol homeostasis and signal transduction, and has been implicated in cancer [44]. Hence, we questioned whether Cav-1 expression is necessary for MT1-MMP expression, PI3K/Akt signaling and Cav-1/MT1-MMP co-localization induced by LSS exposure. MDA-MB-231 cells were transfected by Cav-1 shRNA to suppress Cav-1 expression. Knockdown of Cav-1 resulted in slight inhibition in LSS-induced cell motility (Figure S2), which also suggests that Cav-1 may act in breast cancer metastasis. Next, we further detected the MT1-MMP expression and phosphorylation of PI3K (p85) and Akt (Ser473) after Cav-1 knockdown by Cav-1 shRNA transfection. As shown in Figure 9, Knockdown Cav-1 expression significantly decreased MT1-MMP expression (Figure 9A) and also totally inhibited the activation of PI3K (p85) and Akt (Figure 9B). It has been proposed that Cav-1 is necessary for MT1-MMP expression and PI3K/Akt signaling. Due to trafficking of MT1-MMP to invadopodia is required for the function of Cav-1/MT1-MMP co-localization (association), we next elucidate whether LSS exposure could stimulate Cav-1/MT1-MMP co-localization in cell membranes. MDA-MB-231 cells transfected stably with scrambled shRNA (LKO) or Cav-1 shRNA were kept under static condition as control or subjected to LSS (1.8 dyn) for 1 h. The cells were stained with fluorescence labeled anti-caveolin-1 and DyLight 405 to label the Cav-1 (red) MT1-MMP (blue), respectively. The results of immunocytochemical assay (Figure 10) showed that LSS induced increase in Cav-1/MT1-MMP co-localization (yellow triangular arrows indicated) in scrambled shRNA stably transfected MDA-MB-231 cells (referred as LKO), as compared with the static controls. However, Cav-1/MT1-MMP co-localization completely disappeared in the Cav-1 knockdown cells despite in static condition or LSS exposure. Collectively, these data suggest that Cav-1 is involved in MT1-MMP expression, PI3K/Akt signaling and LSS-induced Cav-1/MT1-MMP association in cell membranes.

### LSS-induced MT1-MMP expression via the activation of PI3K/Akt/mTOR pathway

**Figure 11:**
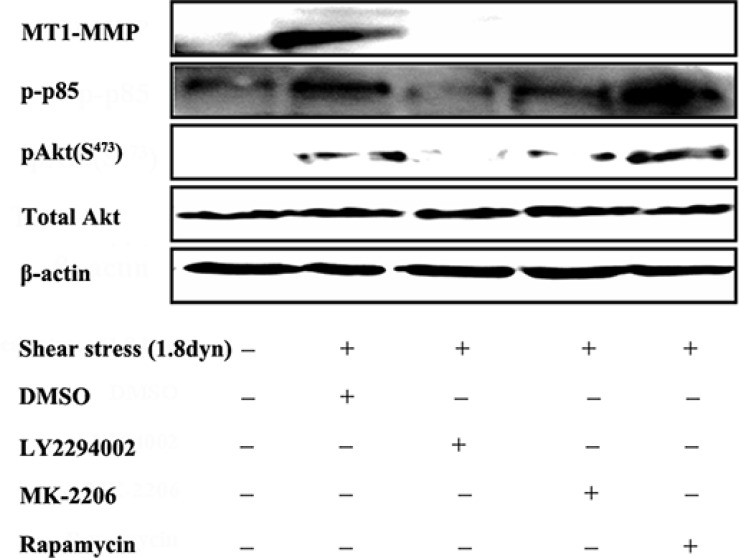
LSS induced MT1-MMP expression through the PI3K/Akt/mTOR pathway MDA-MB-231 cells were kept under static condition as control or subjected to LSS (1.8 dyn) for 1 h. The phosphorylation of PI3K and Akt and MT1-MMP expression were determined by Western blot analysis. Before LSS exposure, the cells were pretreated for 1 h with inhibitors of PI3K (10 μM LY294002), Akt (10 μM MK-2206) or mTOR (10 μM rapamycin).

There is now considerable evidence that MMPs that are intrinsically associated with the plasma membrane due to the presence of a transmembrane domain within their sequence, the so-called membrane type MMPs, represent key components involved in pericellular proteolysis and subsequent cell locomotion and invasion [45, 46]. The prototypical member of this family, MT1-MMP, is crucial in the ECM remodeling by degrading several of its components [47]. Therefore, we attempted to determine whether PI3K/Akt/mTOR pathway participated in the LSS-induced MT1-MMP expression. As shown in Figure 11. Pretreating MDA-MB-231 cells with the specific inhibitors of PI3K (10 μM LY294002), Akt (10 μM MK-2206) and mTOR (10 μM rapamycin) completely abolished the LSS-induced MT1-MMP expression, indicating that the PI3K/Akt/mTOR pathway is required for LSS-induced MT1-MMP expression.

### Cav-1 activation participates in cell polarity and cytoskeleton remodeling

**Figure 12:**
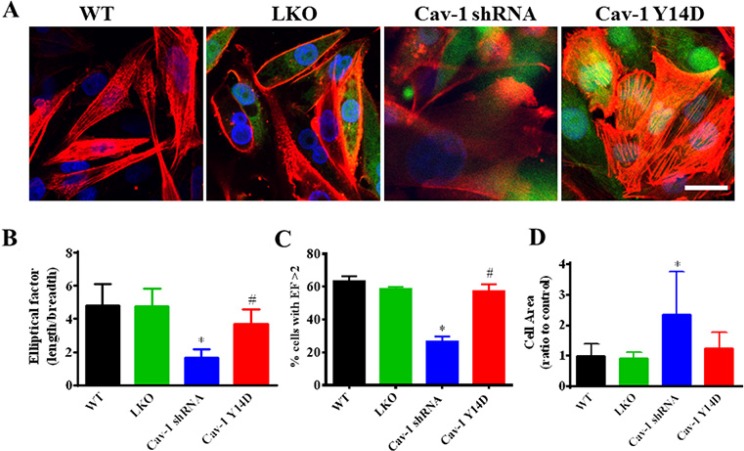
Cav-1 is associated with the polarizated phenotype formation and cytoskeleton rearragment in MDA-MB-231 cells (**A**) Cells were stably transfected with scrambled shRNA (LKO), Cav-1 shRNA or Cav-1 Y14D, and stained with TRTIC-conjugated phalloidin to reveal the morphology of the actin cytoskeleton (red) observed under a confocal microscope. GFP (green), DAPI (blue). (**B**) The elliptical factor (EF) (length/breadth), used as a measure of cell polarization, was calculated for MDA-MB-231 cells (*n =* 15 for each group). (**C**) The percentage of cells with EF > 2 was calculated for each group. (**D**) MDA-MB-231 cell area was calculated for MDA-MB-231 cells (*n =* 15 for each group). All the quantitative data were calculated by ImageJ software (NIH). ^*^p < 0.05 Cav-1 shRNA *vs*. wide type (WT) or LKO; ^#^*p* < 0.05 Cav-1 Y14D *vs*. Cav-1 shRNA.

Metastasis requires increased cell motility, which is a tightly orchestrated process of numerous molecular events, characterized by directed cytoskeletal rearrangements [48]. The ability of a cell to move requires the asymmetrical organization of cellular activities. However, whether Cav-1 participates cancer cell polarity is not well known. Thus, clarifying the underlying roles of Cav-1 in the cytoskeletal reorganization and cell polarity may provide more evidence for breast cancer malignant behaviors. The MDA-MB-231 cells were stably transfected with wild-type (WT), scrambled shRNA (LKO), Cav-1 shRNA, or phosphomimetic Y14D (Cav-1 Y14D), and stained with TRTIC-conjugated phalloidin to reveal the morphology of the actin cytoskeleton (F-actin). We compared the morphological phenotype of cell spread and stress fiber formation by immunofluorescence confocal analysis. As shown in Figure 12A, the Cav-1-knockdown cells displayed a remarkable morphological change with irregular characteristics and a rounded and swelling appearance as compared with the mock cells or WT cells. Most interestingly, the morphology of Cav-1 Y14D (activated Cva-1) transfected cells exhibited a polarized morphology, with an elongated shape, and stress fibers were organized in bundles aligned along the long axis of the cells compared with the Cav-1-knockdown cells. It indicated that sustained activation of Cav-1 in cells could facilitate cell polarity formation. Similar results were obtained by quantitative evaluation of cell polarity, and the Cav-1 Y14D-transfected cells with polarity value above 2 were also significantly higher (Figure 12B and 12C). Moreover, quantitative data showed that suppression of Cav-1 drastically increased cell area (Figure 12D). These results suggest that Cav-1 expression and its activation might augment the elongated morphology (cell polarity) and actin architecture (stress fiber architecture).

### Cav-1 activation enhances breast tumor metastasis

**Figure 13:**
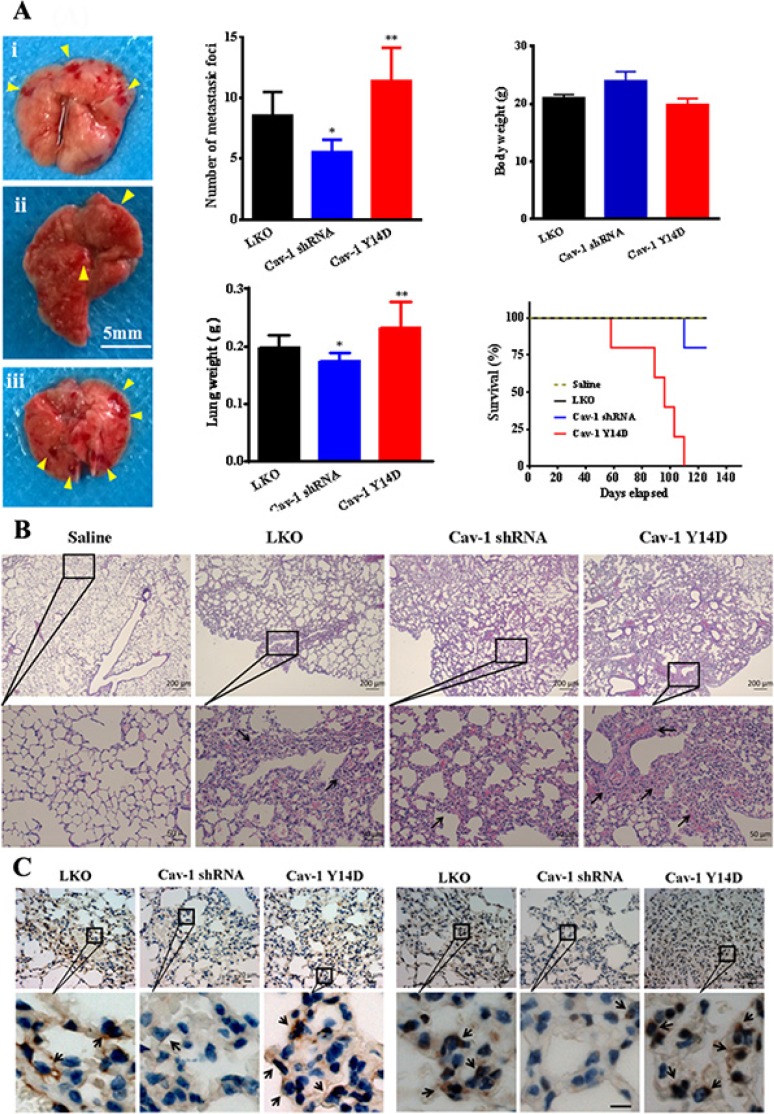
Cav-1 is associated with tumor invasiveness and metastasis *in vivo* MDA-MB-231 cells (2 × 10^6^) transfected with scrambled shRNA (LKO) (i), Cav-1 shRNA (ii) or Cav-1 Y14D (iii) were intravenously injected into the tail vein of BALB/c mice for 8 weeks. Representative images show the appearance of a mice lung (**A**), and hematoxylin and eosin staining of a mice pulmonary section (**B**). Arrows indicate metastatic tumor nodules. Number of metastatic tumor nodules per lung and average lung weight were quantified. For each group, *n =* 6. (**C**) Immunohistochemical analysis of Cav-1 (lift panel) and MT1-MMP (right panel) expression in the lung tumor tissues of metastatic BALB/c mice with the aforementioned MDA-MB-231 cells intravenously injection. Scale bar = 10 μm.

To demonstrate that Cav-1 and its activation indeed contribute to tumor metastasis, stably transfected MDA-MB-231 cells with scrambled shRNA (LKO), Cav-1 shRNA or Cav-1 Y14D, were intravenously injected into tail vein of BALB/c mice. In this model, we evaluated the effect of Cav-1 and its activation on lung metastasis of MDA-MB-231 cells in nude mice. The mice were euthanized and pulmonary colonization was examined for the developed lung metastasis. Results showed that knockdown of Cav-1 effectively suppressed tumor colonization and nodules formation in mice lungs compared with LKO group (Figure 13A). However, endogenous activation of Cav-1 (Cav-1 Y14D group) significantly enhanced tumor dissemination to the lungs, and more metastatic foci were observed. The survival of Cav-1 Y14D group was significantly lower as compared with that of the other groups. Histological examination of the lungs confirmed the above gross findings, upon intravenous injection of transfected MDA-MB-231 cells in nude mice, Cav-1 activation (Cav-1 Y14D) promotes production of massive tumor which had a solid histological appearance and lacked signs of tissue organization. The groups pretreated with saline, LKO or Cav-1 shRNA reduced all the metastatic phenomena stated above (Figure 13B). Furthermore, the enhanced expression of both Cav-1 and MT1-MMP in lung in Cav-1 Y14D group was confirmed by immunohistochemistry (Figure 13C). Stronger staining of Cav-1 and NT1-MMP in tumor lung tissues derived from the Cav-1 Y14D group than those derived from LKO or Cav-1 shRNA groups (black arrows indicated). Taken together, these findings *in vivo* strongly support the idea that Cav-1 activation facilitates to promote metastasis of MDA-MB-231 cells.

## DISCUSSION

**Figure 14:**
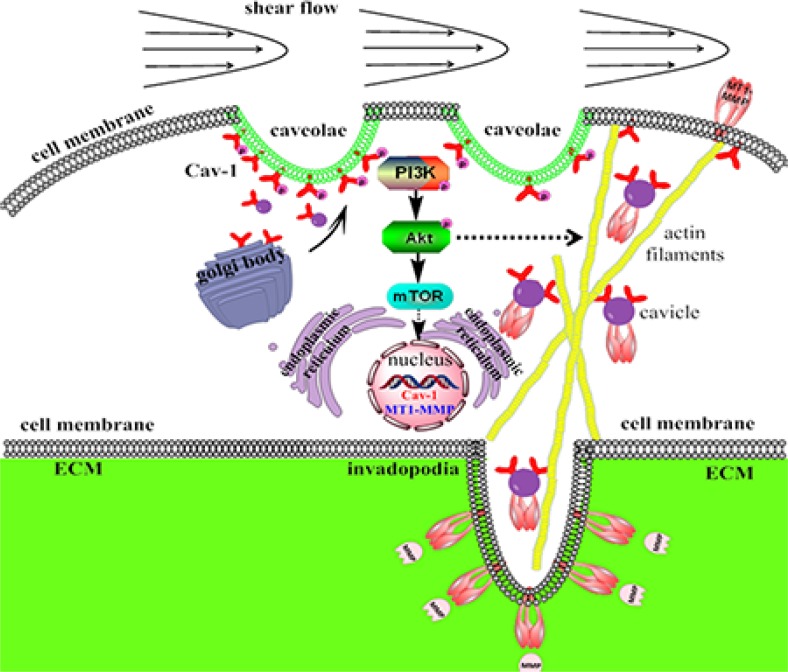
Schematic representation of the signaling pathways regulating human breast carcinoma MDA-MB-231 cells invadopodia formation, motility and metastasis in response to LSS LSS could induce Cav-1 activation and cluster from cytoplasm to cell membrane, and triggered the activation of the PI3K/Akt/mTOR pathway to upregulate MT1-MMP expression and accelerate ECM degradation. In addition, LSS could also remodel cytoskeleton and caveolae trafficking, and promote invadopodia formation and MT1-MMP translocate to the invadopodia, which consequently led to cancer cell hematogenous metastasis.

Identifying biomarkers and regulatory mechanisms is important toward developing diagnostic and therapeutic tools against metastatic cancer. Although the formation of cancer metastasis requires the successful completion of several sequential interrelated steps, cancer cells migrating *in vivo* through the physical barriers of the dense ECM existing in the tumor microenvironment plays an important role in the final metastasis formation. Therefore, a thorough understanding of the mechanisms how the fluid shear stress stimulates cancer metastasis could help researchers develop new therapeutic interventions [49–51]. In the present study, we have characterized a novel mechanism (Summarized in Figure 14) by which LSS induces MDA-MB-231 cell motility and metastasis through the mechanosensitive Cav-1 activation to trigger the downstream PI3K/Akt/mTOR pathways, and increased the expression of Cav-1 and MT1-MMP to facilitate MT1-MMP accumulation in invadopodia. Several lines of evidence support this conclusion. First, Cav-1 could be activated by LSS in an exposure time-dependent manner in MDA-MB-231 cells, and Cav-1 translocation to cell membranes were found under LSS conditions. Second, the LSS-induced cell motility and MT1-MMP expression were significantly inhibited by the specific inhibitors of PI3K, Akt and mTOR; knockdown of Cav-1 also inhibited the phosphorylation of PI3K and Akt. Third, LSS induced co-localization of Cav-1 and MT1-MMP in invadopodia, which was inhibited by Cav-1 shRNA transfection, indicating a synergistic role of Cav-1 and MT1-MMP in the formation of invadopodia for ECM degradation in response to LSS. Finally, cell polarity and stress fiber formation were markedly enhanced by Cav-1 Y14D transfection, a sustained activated Cav-1. In addition, *in vivo* data also demonstrated that overexpression of activated Cav-1 in MDA-MB-231 cells promoted the lung metastasis.

The PI3K/Akt/mTOR pathway has been shown to involve in the modulation of cell growth, survival, adhesion and migration in response to chemical stimuli [52, 53]. However, the role of this signaling pathway in cancer cell motility and metastasis in response to mechanical stimuli remains unclear. Our present study demonstrated for the first time that LSS induces sustained phosphorylation of PI3K and Akt in MDA-MB-231 cells, with Akt being upstream from mTOR. Pretreatment with the specific inhibitors of PI3K, Akt and mTOR inhibited the LSS-induced cell motility and MT1-MMP expression, indicating the importance of the PI3k/Akt/mTOR pathway in modulating the malignant cellular behaviors during hematogenous metastasis.

Cav-1 is a multifunctional scaffolding protein with multiple binding partners that associates with cell surface caveolae. In addition, Cav-1 could also function as a membrane adaptor. For example, caveolin-1 links the integrin α-subunit to the c-Src kinase pathway and subsequently to the mitogen-activated protein kinase pathway to promote cell cycle progression [54]. Recently, numerous literatures have reported that Cav-1 regulated multiple cancer-associated cellular processes, and its expression is a marker that predicts poor cancer patient prognosis [55, 56]. Here, we showed that LSS exposure increased the Cav-1 and MT1-MMP expression and induced Cav-1/MT1-MMP co-localization at cell membranes, especially at invadopodia. It is possible that the caveolae internalizes and actively traffics MT1-MMP to the invadopodia. Cav-1 may also coordinately promote the MT1-MMP transport in the same caveolae compartment. Reasonably, we could deduce that LSS could induce the translocation of Cav-1 from the cytoplasm to the cell membrane, increase the caveolae formation preferentially at the luminal surface.

The lipid raft disruption significantly blocking the gelatin degradation further demonstrated that MT1-MMP accumulation at invadopodia could accelerate cancer cell motility or metastasis. Invadopodia are actin-rich cell membrane projections used by invasive cells to penetrate the basement membrane. Invadopodia number as well as its stability is critical for efficient ECM degradation [57]. LSS-enhanced cancer cell motility partly attributes to more invadopodia formation. However, the underlying molecular mechanisms remain poorly understood. Here, we uncover a new role for Cav-1, which could be activated by LSS to trigger PI3K/Akt/mTOR pathway, then leading to enhance invadopodia-mediated matrix degradation and MT1-MMP translocation. To further test this, we found that knockdown of Cav-1 significantly inhibited MT1-MMP expression and the phosphorylation of PI3K and Akt. These data demonstrated that Cav-1 may be critical in mechanosensing and subsequent mechanosensitive cell signaling.

Cell polarization is the first step required for cell migration and directional movement. Recent reports suggest that augmented Cav-1 expression has been associated with enhanced metastatic potential, although the role of Cav-1 in cell migration is still controversial [44, 58, 59]. In our study, we found that the Cav-1 activated cells (Cav-1 Y14D group) exhibited typical fibroblast-like phenotype with actin filaments bundled into thick contractile stress fibers at the ventral cell surface, whereas Cav-1-deficient cells (Cav-1 shRNA group) showed cortical thin bundles of actin filaments. Quantitative analysis also indicated that Cav-1 knockdown deregulated MDA-MB-231 cell polarization. The phenotype changes suggested alterations in the activity of the GTPases of the Rho family, as they are the principal regulators of polarity and cytoskeletal rearrangements [60, 61]. Collectively, the evidence demonstrated that Cav-1 and its activation are required for the establishment of a polarized and elongated morphology.

The ability to proliferate at distant sites is essential for the establishment of early metastatic lesions. We further confirmed the role of Cav-1 by *in vivo* studies using the lung metastasis model. The dramatic effects of activated Cav-1 on cell metastasis and animal survival were evident in the development of metastatic foci in the lungs. The incidence of lung metastasis in Cav-1 shRNA group decreased notably, which are also well in agreement with the HE staining data. Furthermore, immunohistochemical assays in the lungs demonstrated that both Cav-1 and MT1-MMP were overexpressed in Cav-1 Y14D group. Cav-1-dependent MT1-MMP trafficking and Cav-1 activation would promote metastatic foci formation. Taken together, the results from *in vivo* study demonstrate for the first time that Cav-1 is an important regulator of tumor growth and metastasis.

In summary, this study provides evidence that Cav-1 and its activation play critical roles in promoting LSS-induced invadopodia formation and tumor metastases. LSS exposure was found to induce mechanosensitive Cav-1 activation and PI3K/Akt/mTOR signaling cascade, which increase the MT1-MMP expression and translocation, cytoskeleton reorganization, invadopodia formation and ECM degradation, leading to promotion of tumor cell motility and metastasis. These findings provide a new insight into the role of Cav-1 in cancer cell metastasis that might contribute to the development of new therapeutic strategies for breast cancer treatment.

## MATERIALS AND METHODS

### Antibodies and reagents

Monoclonal antibody against the caveolin-1, Akt, phospho-Akt, p85, and phospho-p85 were purchased from Cell Signaling Technology (Beverly, MA, USA). Texas Red-labeled goat anti-rabbit secondary polyclonal antibody and FITC-labeled goat anti-mouse secondary polyclonal antibody were from ABclonal Technology (Cambridge, MA, USA). Horseradish peroxidase (HRP)-coupled goat anti-rabbit secondary polyclonal antibody and anti-actin were obtained from ZSGB-BIO (Beijing, China). Rabbit monoclonal antibody anti-pY14-caveolin-1 and DyLight 405-labeled goat anti-mouse secondary polyclonal antibody were purchased from Santa Cruz Biotechnology (Texas, USA) and Abbkine (Redlands, CA, USA), respectively. Alexa Fluor 488-conjugated cholera toxin subunit B (CTxB), calcein-AM, and fluorescein isothiocyanate (FITC)-conjugated gelatin were from Invitrogen Life Technologies (Carlsbad, CA, USA). Inhibitors of PI3K (LY294002), Akt (MK-2206), Src (PP2), FAK (PF573228) and mTOR (rapamycin) were purchased from Abmole Bioscience (Houston, TX, USA). Water-soluble cholesterol and methyl-β-cyclodextrin (MBCD), cholesterol, and TRTIC-conjugated phalloidin were supplied by Sigma–Aldrich (St. Louis, MO, USA). All other chemicals and solvents if not mentioned were of analytical grade and used as received without additional purification.

### Cell culture

Human breast cancer cell line of MDA-MB-231 (ATCC number HTB-26) was obtained from American Type Culture Collection (ATCC, Manassas, VA, USA) and grown in L15 culture medium supplemented with 10% fetal bovine serum (FBS) (Hyclone, UT, USA), 100 mg/ml streptomycin and 100 units/ml penicillin in a humidified incubator at 37°C and without CO_2_ atmosphere. Prior to following experiments, cells were detached from 25-cm^2^ culture flasks with 0.25% trypsin in phosphate buffered saline (PBS) (pH 7.4).

### Plasmids and transfection

The transfection has been performed as described previously [22]. The constitutive activated Cav-1 (Cav-1 Y14D) expression plasmid, Cav-1 knockdown Cav-1 shRNA plasmid (Cav-1 shRNA1 and Cav-1 shRNA2), control empty and scrambled shRNA vectors were obtained from Cyagen Biosciences (Santa Clara, CA, USA). Stable transfections of the Cav-1 Y14D expression plasmid or Cav-1 shRNA plasmid were generated by culturing MDA-MB-231 cells in a 6-well plate until they reached 40% confluence. Lipofectamine LTX reagent (Invitrogen, Carlsbad, CA, USA) and Cav-1 Y14D or Cav-1 shRNA plasmid were used to transfect the cells in the absence of serum. After 24 h, the medium was replaced with a culture medium containing 10% FBS. Approximately 3–4 days after the beginning of the transfection, the cells were digested with 0.05% trypsin; the cell suspensions were seeded in 75-cm^2^ culture flasks and cultured for a few days with drug selection. The stable transfectants were pooled, and the expression of Cav-1 protein in the transfectants was confirmed by western blotting.

### Shear flow apparatus

A rectangular parallel plate perfusion chamber [23–25], designed and presented by professor Jeng-Jiann Chiu Lab in the National Health Research Institutes of Taiwan, was used for fluid shear stress exposure (see Figure S1). This system comprises a transparent polymethylmethacrylate plate, two silastic rubber gaskets, and a standard glass coverslip. The coverslip with near 90% confluent monolayer of MDA-MB-231 cells was mounted over the groove with the cells facing the inside, and an approximate 200 μm high gap was formed over the MDA-MB-231 cells. The wall shear stress (*τ_w_*) is related to the volumetric flow rate (Q) by *τ_w =_* 6 *μQ/w(h)*^2^, where μ is the fluid viscosity, *w* is the width of the flow field and *h* is the height. The shear stress can be controlled through flow rate of *Q*. The chamber was placed on the stage of an inverted microscope.

### Scratch motility assay

MDA-MB-231 cells motility was analyzed by a scratch wound assay. MDA-MB-231 cells (2 × 10^5^ cells/ml) were plated in a coverslip and grown to confluence in serum-containing medium. The monolayer was scratched with a pipette tip, washed with PBS to remove floating cells and photographed (0 h). The cells were subjected to shear flow exposure for 1 h, and continue to culture for 24 or 48 h in culture dishes. No fluid shear stress exposed sample was referred as the control (static culture). All the samples were stained by calcein-AM (Invitrogen). The closure into the scratched area was photographed under an inverted fluorescence microscope (TE-2000U, Nikon, Japan) and wound area was measured by ImageJ software and plotted as the fold of wound closure relative to control (static culture). More than five random fields were selected and mean value per field was expressed.

### Immunofluorescence microscopy

Immunofluorescence was conducted as previously described [25, 26]. Briefly, MDA-MB-231 cells were washed with PBS and fixed in 4% paraformaldehyde for 15 min and followed by washing three times in Tris buffered saline with Triton (TBST, 20 mM Tris, 150 mM NaCl, 0.1% Triton X-100) for 10 min, and incubating with blocking buffer (TBST with 5% bovine serum albumin) for 2 h at room temperature. For labeling membrane lipid rafts with CTxB, the treated MDA-MB-231 cells were fixed in 4% paraformaldehyde for 20 min at room temperature, and incubated with Alexa Fluor 488-conjugated CTxB antibody (1:200) for 30 min. For Cav-1staining analysis, the cells were incubated with anti-caveolin-1 monoclonal antibody for 1 h at room temperature, followed by incubation with Texas Red-labeled secondary antibody for 1 h. After washing with TBST for several times, cell nuclei were stained by DAPI, then the fluorescent images were obtained using a confocal laser scanning microscope (Nikon A1, Tokyo, Japan). For co-localization analysis of Cav-1 and p85 (a regulatory subunit of PI3K), MDA-MB-231 cells were firstly stained as aforementioned method, then incubated with mouse anti-human p85 monoclonal antibody (1:200) for 1 h. After washing in TBST with gentle shaking, the cells were stained with FITC-labeled goat anti-mouse IgG (1:1000) for 1 h at room temperature. The cell nuclei were also stained by 4′, 6-diamidino-2-phenylindole dihydrochloride (DAPI) (Beyotime, Beijing, China), and then were washed three times in TBST for 10 min again. The samples were observed with a confocal laser scanning microscope (Nikon A1, Tokyo, Japan).

### Invadopodia assay and gelatin degradation

FITC-conjugated gelatin-coated coverslips were prepared as described previously [10, 27, 28]. MDA-MB-231 cells (5 × 10^4^) were cultured for 7 h on coverslips coated with FITC-conjugated gelatin. The cells were kept under static condition as control or subjected to low shear stresses (1.8 or 4.0 dynes/cm^2^) for 1 h, and continue to culture for 12 or 24 h in a culture dish. To detect invadopodia, the cells were stained with TRTIC-conjugated phalloidin (1:2000) and DAPI, before being observed under a confocal microscope (Nikon A1, Tokyo, Japan). Invadopodia were identified as morphologically characteristic actin-cortactin dots found at the bottom of the cell in the XZ dimension. Foci of degraded matrix were visible as dark dot-like areas that lack fluorescence and appear as ‘holes’ in the bright fluorescent gelatin matrix [29].

### Western blotting

The Western blotting was conducted as described previously [8]. Proteins (20 μg) from cell homogenates were separated on a 10% SDS-polyacrylamide gel and electrotransfer to polyvinylidene fluoride membranes (Millipore, Bedford, MA, USA). Each membrane was washed with Tris-buffered saline Tween-20 [TBST: 10 mM Tris-HCl (pH 7.6), 150 mM NaCl, and 0.05% Tween-20], blocked with 5% skim milk power for 1 h, and incubated with appropriate primary antibodies at dilutions recommended by the supplier. Membranes were washed in TBST and probed with an appropriate HRP-conjugated secondary antibody (goat anti-rabbit or goat anti-mouse IgG). The oxidase components were detected by chemiluminescence (BeyoECL Plus) (Beyotime, Jiangsu, China).

### Quantitative PCR (qPCR)

Total RNA was isolated using the Trizol reagent (Invitrogen). For *q*PCR analysis, it was performed in accordance with the manufacturer's instructions using SYBR Premix Ex Taq II (Tli RNaseH Plus) (Takara). The master mix containing Takara Ex Taq HS, dNTP mixture, Mg^2+^, Tli RNaseH and SYBR Green I. One microliter of reverse transcription reaction was used for a total 25 μl quantitative PCR reactions. Relative mRNA levels of each gene were analyzed according to the comparative C_*t*_ method and were normalized to the expression of GAPDH and are plotted as means ± standard deviations of duplicate PCRs. The following sets of primers were used in the PCR amplification: GAPDH (forward, 5′-GAGAGACCCTCACTGCTG-3′; reverse, 5′-GATGGTACATGACAAGGTGC-3′). Cav-1 (forward, 5′-CCAGCTTCACCACCT TCACT-3′; reverse, 5′-CACAGACGGTGTGGACGTAG-3′). MT1-MMP (forward, 5′-CCTTGGACTGTCAGGAATGAGG-3′; reverse, 5′-TTCTCCGTGTCCATCCACTGGT-3′). For all the qPCR experiments, the correlation coefficient and amplification efficiency in the reaction using each primer are 0.97–1.02 and more than 97.9%, respectively.

### Labeling cells with CTxB

For labeling membrane lipid rafts with Alexa Fluor 488-conjugated CTxB [26, 27], the MDA-MB-231 cells were fixed in 4% paraformaldehyde for 20 min at room temperature, and incubated with Alexa Fluor 488-conjugated CTxB (5 μg/ml) for 30 min at 4°C. After washing three times with PBS, cell nuclei were stained by DAPI. Fluorescent images were captured by a confocal laser scanning microscope (Nikon A1, Tokyo, Japan).

### Cholesterol depletion and replenishment experiment

Membrane cholesterol was depleted and replenished, as previously described [25, 26, 30]. MDA-MB-231 cells were treated with MBCD (10 μM) at 37°C for 60 min. Subsequently the cells were washed with PBS and incubated with medium containing water-soluble cholesterol (84 μg/ml) for 30 min. The cells were exposed to low shear stress (1.8 dynes/cm^2^) for 1 h. The activation of PI3K, Akt and Cav-1 and ECM degradation were analyzed by Western blot or immunofluorescence microscopy mentioned above.

### Animal models

For the experimental lung metastasis assay [15], MDA-MB-231 cells (2 × 10^6^) transfected stably with scrambled shRNA (LKO), Cav-1 shRNA or Cav-1 Y14D were intravenously injected into the tail vein of 4-week-old BALB/c mice, which were then monitored for 8 weeks. All dissected lungs were embedded with paraffin, and tissue sections were stained with hematoxylin and eosin. Metastatic tumor colonies were counted under a microscope. All animals were cared for in a specific pathogen-free room and treated in accordance with the animal care protocol approved by the Animal Ethics Committee of University of Electronic Science and Technology of China (approval number UESTC-AEC20140083, Feb. 26, 2014).

### Immunohistochemistry

Immunohistochemical assays were performed using a DAB Color Developing Reagent Kit (Beyotime, Jiangsu, China). Paraffin-embedded lung sections were de-paraffinized, rehydrated, and autoclaved to retrieve antigens. Endogenous peroxidase activity was blocked by incubation in 3% H_2_O_2_ in methanol for 10 min at room temperature. Sections were incubated overnight at 4°C with a 1:100 dilution of anti-caveolin-1 or anti-MT1-MMP monoclonal antibodies in PBS. For the negative control, the rabbit or mouse serum was used as the control antibodies, not without first antibody incubation. After rinsing in PBS, the sections were incubated with horseradish peroxidase-coupled goat antibody. The diaminobenzidine reaction was performed using a liquid diaminobenzidine substrate-chromogen system. After the tissues were rinsed in PBS, they were counterstained with hematoxylin and eosin. Immunolabeled sections were examined by light microscopy and processed with Adobe Photoshop software [31].

### Statistical analysis

All data were expressed as mean ± standard deviation (SD). All values were obtained from at least three independent experiments. Results were analyzed using GraphPad Prism Software version 6.0 (GraphPad Software Inc., San Diego, CA). Statistical analysis was performed using one-way ANOVA, and *p* < 0.05 was considered statistically significant.

## SUPPLEMENTARY MATERIALS FIGURES


