# Correction: Mechanosensitive caveolin-1 activation-induced PI3K/Akt/mTOR signaling pathway promotes breast cancer motility, invadopodia formation and metastasis *in vivo*

**DOI:** 10.18632/oncotarget.26065

**Published:** 2018-08-24

**Authors:** Hong Yang, Liuyuan Guan, Shun Li, Ying Jiang, Niya Xiong, Li Li, Chunhui Wu, Hongjuan Zeng, Yiyao Liu

**Affiliations:** ^1^ Department of Biophysics, School of Life Science and Technology, University of Electronic Science and Technology of China, Chengdu 610054, Sichuan, P.R. China; ^2^ Center for Information in Biomedicine, University of Electronic Science and Technology of China, Chengdu 610054, Sichuan, P.R. China

**This article has been corrected:** The correct author affiliation is given below:

^1^ Department of Biophysics, School of Life Science and Technology, University of Electronic Science and Technology of China, Chengdu 610054, Sichuan, P.R. China

Original article: Oncotarget. 2016; 7:16227-16247. https://doi.org/10.18632/oncotarget.7583

