# Supplementary material for: Mechanosensitive caveolin-1 activation-induced PI3K/Akt/mTOR signaling pathway promotes breast cancer motility, invadopodia formation and metastasis in vivo
Source: Oncotarget. 2016 Feb 22;7(13):16227–47. doi: 10.18632/oncotarget.7583 (PMC4941310; doi:10.18632/oncotarget.7583)
Supplement: Supplementary file 1 [file oncotarget-07-16227-s001.pdf]

Mechanosensitive caveolin-1 activation-induced PI3K/Akt/mTOR signaling pathway promotes breast cancer motility, invadopodia formation and metastasis *in vivo*

Supplementary Materials

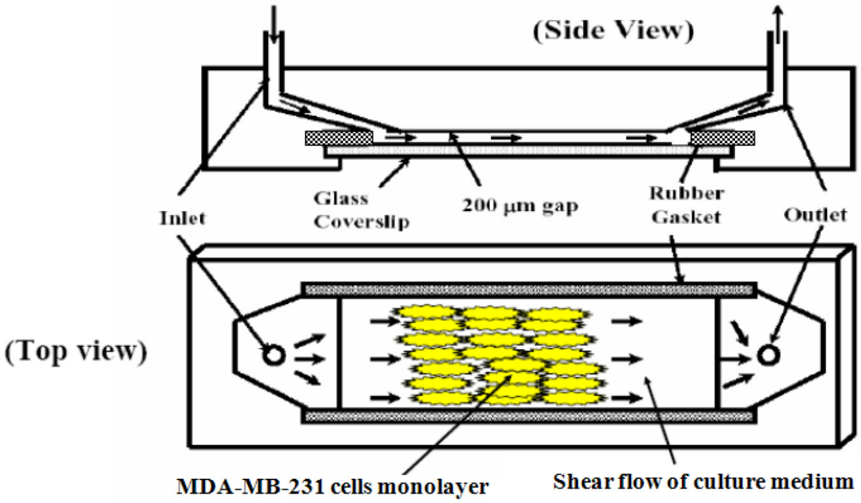

Supplementary Figure S1: Cross-sectional diagram of parallel flow chamber system for shear stress exposure. The coverslip with near 90% confluent monolayer of MDA-MB-231 cells was mounted over the groove with the cells facing the inside, and an approximate 200 μm high gap was formed over the MDA-MB-231 cells.

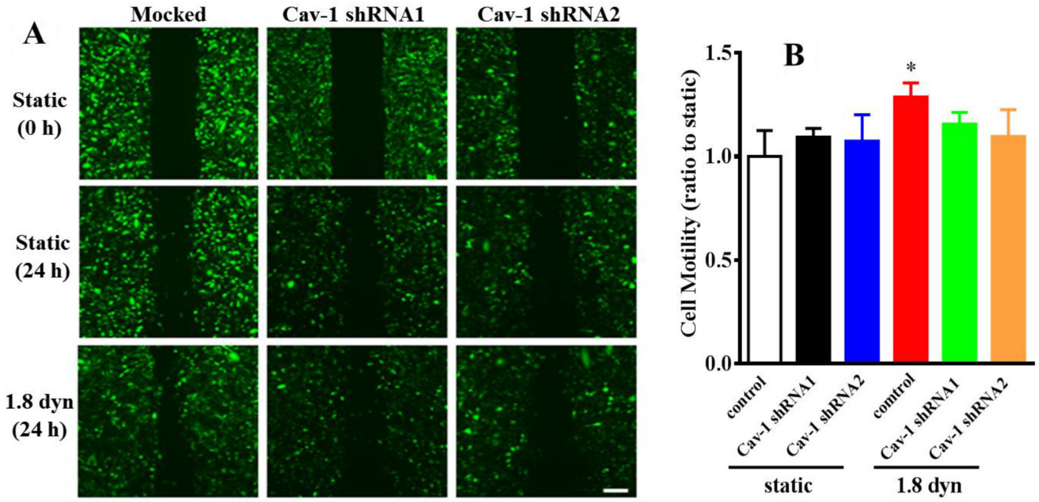

Supplementary Figure S2: Downregulation of cav-1 expression decreased the low shear stress-induced cell motility by wound healing assay. MDA-MB-231 cells was stably transfected with shRNA targeting caveolin-1 (cav-1 shRNA1 or cav-1 shRNA2), and kept under static condition as control or subjected to low shear stress for 1 h. The monolayers were wounded with a pipet tip, and stained by calcein-AM. The images were recorded at 0 and 24 h post-wounding (**A**). The wounded area was measured with the ImageJ software (NIH, USA), and the cell motility was quantified by calculating wound closure from six microscopic fields for each sample (**B**) and was expressed as mean ± SD. *$p < 0.05$ control at 1.8 dyn vs. Cav-1 silencing at 1.8 dyn.
